# Piceatannol, a Dietary Polyphenol, Alleviates Adipose Tissue Loss in Pre-Clinical Model of Cancer-Associated Cachexia via Lipolysis Inhibition

**DOI:** 10.3390/nu14112306

**Published:** 2022-05-31

**Authors:** Jonathan C. Kershaw, Bennett D. Elzey, Xiao-Xuan Guo, Kee-Hong Kim

**Affiliations:** 1 Department of Food Science, Purdue University, West Lafayette, IN 47907, USA; jkersha@bgsu.edu; 2 Department of Public and Allied Health, Bowling Green State University, Bowling Green, OH 43403, USA; 3 Purdue Center for Cancer Research, Purdue University, West Lafayette, IN 47907, USA; belzey@iupui.edu; 4 Institute of Quality Standard and Testing Technology for Agro-Products, Chinese Academy of Agricultural Sciences, Beijing 100081, China; guoxiaoxuan@caas.cn

**Keywords:** adipose, cachexia, cancer, lipolysis, piceatannol

## Abstract

Cancer-associated cachexia (CAC) is the nutrition-independent loss of lean muscle and adipose tissues, and results in reduced chemotherapy effectiveness and increased mortality. Preventing adipose loss is considered a key target in the early stages of cachexia. Lipolysis is considered the central driver of adipose loss in CAC. We recently found that piceatannol, but not its analogue resveratrol, exhibits an inhibitory effect on lipolysis. The objective of this study was to investigate the role of piceatannol in cancer-associated lipolysis and cachexia-induced weight loss. Cancer cell-induced lipolysis in adipocytes was stimulated using cancer-conditioned media (CCM) or co-culture with human pancreatic cancer cells and the cachexia-associated cytokines TNF-α and interleukin-6 in 3T3-L1 adipocytes. C26 colon carcinoma-bearing mice were modeled using CAC *in vivo*. Piceatannol reduced cancer-associated lipolysis by at least 50% in both CCM and cytokine-induced lipolysis in vitro. Further gene and protein analysis confirmed that piceatannol modulated the stability of lipolytic proteins. Moreover, piceatannol protected tumor-bearing mice against weight-loss in early stages of CAC largely through preserving adipose tissue, with no effect on survival. This study demonstrates the use of a dietary compound to preserve adipose in models of early stage CAC and provides groundwork for further investigation of piceatannol or piceatannol-rich foods as alternative medicine in the preservation of body fat mass and future CAC therapy.

## 1. Introduction

The uncontrolled wasting induced by cancer-associated cachexia (CAC) results in functional impairment, decreased chemotherapy effectiveness, lower quality of life, and poorer survival outcomes [1,2]. CAC is the direct cause of death in up to 20% of all cancer mortalities [1,3]. CAC is characterized by an ongoing loss of adipose and lean muscle, which cannot be overcome with nutritional interventions [4,5]. Although the causes of CAC are not fully understood, dysregulated energy balance likely plays a significant role [6,7,8]. Despite the lack of a universal treatment strategy, managing symptoms of CAC can increase survival and improve quality of life [9,10].

While CAC treatments have historically focused on minimizing lean muscle loss, recent studies indicate an increasingly important role of adipose atrophy. Of note, adipose loss precedes muscle loss and inversely correlates with survival in cancer patients [6,11,12,13]. Preventing white adipose tissue (WAT) depletion is considered an important strategy in overall body weight preservation in CAC [6]. Lipolysis, the hydrolysis of stored triglycerides (TG), is primarily responsible for adipose loss in CAC rather than factors such as impaired lipogenesis or adipogenesis [14,15,16]. Furthermore, targeting the principle and rate-limiting lipolytic enzyme, adipose triglyceride lipase (ATGL), preserves both muscle and adipose tissue in mice [17]. Of note, ATGL accounts for up to 70–85% of adipocyte lipolysis [18,19]. Interestingly, pharmacological inhibition of ATGL reduced weight gain and insulin resistance in mouse models of obesity [20], thus providing evidence that lipolysis inhibition is unlikely to result in weight gain. Cancer-induced changes in circulating factors, such as IL-6 and tumor necrosis factor-α (TNF-α), contribute to increased adipose lipolysis in CAC, possibly through modulating ATGL [15,17,21,22]. Due to the established role of cancer-secreted factors in CAC, cancer conditioned media (CCM) has been used for mechanistic studies of the syndrome [6,8,23,24,25].

Piceatannol, a naturally occurring resveratrol analogue, is found in a variety of food sources and has numerous reported health benefits, including anti-cancer, anti-inflammatory, and anti-adipogenic properties [26,27,28]. Additionally, we and others recently found that piceatannol inhibited lipolysis in vitro and *in vivo* [29,30]. Unlike resveratrol, we found that piceatannol induced degradation of lipolytic proteins in adipocytes, including ATGL, its co-activator comparative gene identification-58 (CGI-58), and perilipin1 (PLIN1), in an autophagy-dependent manner. However, whether piceatannol’s anti-lipolytic properties influence CAC-associated lipolysis is unknown.

The aim of this study was to investigate the protective role of piceatannol in models of CAC. Because piceatannol reduces lipolysis via degradation of the ATGL-CGI-58-perilipin lipolytic protein cluster in adipocytes and ATGL plays an important role in CAC adipose loss, we postulated that it would prevent CAC-induced lipolysis in vitro and weight loss *in vivo*. CAC-associated lipolysis was stimulated by exposing 3T3-L1 adipocytes to (1) established human pancreatic cancer cell lines, a cancer type highly associated with CAC, using CCM and co-culture (PANC-1 and AsPC-1, respectively); and (2) lipolytic CAC-associated cytokines TNF-α and IL-6 [1]. We demonstrated that piceatannol blocked CAC-associated lipolysis in all *in vitro* models tested. Furthermore, piceatannol slowed tumor-induced weight-loss and adipose atrophy in colon-26 (C26) carcinoma-bearing mice. We provide novel evidence that a dietary compound, piceatannol, may possess anti-CAC properties.

## 2. Materials and Methods

### 2.1. Cell Culture

Murine 3T3-L1 preadipocytes were purchased from American Type Culture Collection (Manassas, VA, USA). Briefly, pre-adipocytes were maintained in DMEM supplemented with 10% fetal calf serum (FCS), 1% sodium pyruvate, and 1% penicillin-streptomycin. Cells were passaged prior to reaching 80% confluence. To differentiate, preadipocytes were grown to confluence; two days post-confluence (designated as day 0), preadipocytes were treated with differentiation media consisting of 10% fetal bovine serum (FBS), 0.5 mM 3-Isobutyl-1-methylxanthine (IBMX), 1 µM dexamethasone, and 167 nM or 334 nM insulin. On day 2, media was removed and replaced by 10% FBS with 167 nM or 334 nM insulin, with or without 2 µM rosiglitazone to maximize lipid droplet growth. On day 4, differentiation media was replaced with standard media, with or without insulin, and maintained until maturity. PANC-1 human pancreatic carcinoma cells and NIH-3T3 fibroblast cells were maintained in DMEM supplemented with 10% FBS and 10% FCS, respectively, and passaged prior to reaching confluence. Mature adipocytes were used for all experiments. Unless otherwise noted, serum-free media (SFM) refers to DMEM supplemented with 1% BSA, 1% sodium pyruvate, and 1% penicillin-streptomycin. Conditioned media was obtained by culturing confluent PANC-1 cells (CCM) or NIH-3T3 cells (fibroblast-conditioned media, FCM, control condition) overnight in SFM. Media was collected, centrifuged at 4750× *g* for 5 min, aliquoted, and stored at −20 °C until use. For co-culture experiments, AsPC-1 human pancreatic cancer cells were grown on 6-well plate transwell inserts (Corning, Lowell, MA, USA), then co-cultured with either HPDE6 human pancreatic duct epithelial cells or 3T3-L1 cells for the indicated time. 

### 2.2. In Vitro Model of CAC-Induced Lipolysis

To simulate CAC-induced lipolysis, adipocytes were first pre-treated with the stimulus (e.g., TNF-α, IL-6, or CCM) for 16 h, based on the observation of TNF-α’s maximum effect on lipolysis [31]. Adipocytes were then co-treated with stimulus and piceatannol (Enzo Life Sciences, Farmingdale, NY, USA) for 8 h, based on optimal piceatannol stability (data not shown). Commercial kits were used to measure glycerol release (Sigma, St. Louis, MO, USA) and free fatty acids (Cayman Chemical, Ann Arbor, MI, USA), as indicators of lipolysis.

### 2.3. Western Blot

Cells were collected by scraping in ice-cold phosphate buffered saline (PBS). After a light centrifugation, the PBS supernatant was removed and lysis buffer (100 mM Tris-HCl, pH 8.0, 100 mM NaCl, 0.5% NP-40) with protease inhibitors was mixed with the cell pellet and allowed to sit on ice for 10 min. After centrifugation, the supernatant was collected, and protein concentration was measured using the Bradford method. Protein was loaded onto SDS-PAGE gels and separated by electrophoresis. Proteins were then transferred to either a nitrocellulose or polyvinylidene fluoride membrane and incubated with the antibody of interest. Antibodies against β-actin and rabbit secondary antibody were purchased from Santa Cruz Biotechnology (Dallas, TX, USA). Antibody against comparative gene identification-58 (CGI-58) was obtained from Biovision Inc. (Milpitas, CA, USA). Mouse secondary antibody was obtained from Jackson ImmunoResearch Laboratories, Inc. (West Grove, PA, USA). Antibodies against adipose triglyceride lipase (ATGL), and phospho-hormone sensitive lipase (HSL) was obtained from Cell Signaling Technology (Danvers, MA, USA). STAT3 and phospho-STAT3 antibodies were obtained from R&D Systems (Minneapolis, MN, USA). Ponceau S staining was used to confirm transfer efficiency. The membrane was then incubated with the corresponding horseradish peroxidase-linked secondary antibody. Proteins were visualized using enhanced chemiluminescence reagents.

### 2.4. Isolation of Total RNA and PCR

Total RNA was extracted using TRIzol reagent, according to the manufacturer’s protocol. Isolated RNA (2 μg/μL) in combination with M-MuLV reverse transcriptase kit was used to synthesize cDNA, then amplified by PCR. Quantitative PCR was performed using diluted cDNA, primers of interest, and SYBR green Taq mixture in the StepOne real-time PCR system (Applied Biosystems, Foster City, CA, USA). The primer sequences are as follows: *β-actin* (forward, 5′-AGA TGA CCC AGA TCA TGT TTG AGA-3′; reverse, 5′-CAC AGC CTG GAT GGC TAC GT-3′); *ATGL* (forward, 5′-GAG ACC AAG TGG AAC ATC-3′; reverse, 5′-GTA GAT GTG AGT GGC GTT-3′); *Perilipin* (forward, 5′-TGC TGG ATG GAG ACC TC-3′; reverse, 5′-ACC GGC TCC ATG CTC CA-3′); *CGI-58* (forward, 5′-GGT TAA GTC TAG TGC AGC-3′; reverse, 5′-AAG CTG TCT CAC CAC-3′); *HSL* (forward, 5′-ACT CAG ACC AGA AGG CAC TA-3′; reverse, 5′-TAG TTC CAG GAA GGA GTT GA-3′).

### 2.5. Cell Viability

Thiazolyl blue tetrazolium bromide (MTT) was dissolved in PBS at a concentration of 5 mg/mL and filtered. MTT solution was added to fresh SFM at a concentration of 100 µL/mL and cultured for one hour at 37 °C with 3T3-L1 cells in a 24-well plate following experimental treatment. The medium was aspirated and resulting formazan crystals were dissolved in 300 µL of DMSO. Following an additional 1:1 dilution with DMSO, the solution from each well was measured spectrophotometrically at 595 nm using a microplate reader.

### 2.6. Animal Experiment

Mice studies were approved by the Purdue Animal Care and Use Committee (protocol no.: 112000342). Male BALBc mice (7 weeks old) (Jackson Laboratories, Bar Harbor, ME, USA) were fed a standard 18% protein chow diet. After a 2-week adjustment period, mice were randomly divided into three groups: non-tumor bearing (NT, *n* = 4), tumor-bearing (TB, *n* = 9), and piceatannol-treated tumor bearing (PTB, *n* = 9). Mice received an injection of either PBS in the NT group or 5 × 10^5^ murine C26 colorectal carcinoma cells in the TB and PTB groups in the inguinal brown adipose tissue (day 0), an established model of CAC [32]. Piceatannol (4 mg/mL) was dissolved in 30% PEG-400, 1% polysorbate-80, and 1% ethanol to create a treatment stock solution. Stock solutions were stored at 4 °C and prepared fresh twice per week. After tumors became palpable (day 5), tumor-bearing mice received daily intraperitoneal (*i.p.*) injections of either piceatannol (20 mg/kg BW) or vehicle alone. This dose of piceatannol was chosen based on our previous observations that *i.p.* administration of piceatannol higher than 10 mg/kg BW was needed to exhibit significant reduction of lipolysis in lean mice [30]. Tumor volume was measured using calipers. Body score was determined by a trained technician, where 3 indicated a mouse in optimal condition, 2 indicated under-conditioning and prominent appearance of the skeletal structure, and 1 indicated advanced muscle and fat wasting and very prominent appearance of the skeletal structure [33]. Individual mice were euthanized when weight loss exceeded 20% of initial body weight. To determine the effect of piceatannol on survival, mice continued to receive treatment until euthanasia criteria was met. Prior to euthanasia, body composition was determined using Echo-MRI (Echo Medical Systems, Houston, TX, USA). Mice were then euthanized via asphyxiation and cervical dislocation. Data shown are from day 12, when the first mouse met euthanasia criteria.

### 2.7. Statistical Analysis

Statistical analysis was conducted using Minitab 17 (Minitab Inc., State College, PA, USA). Student’s t-test or one-way ANOVA followed by Tukey’s post-hoc difference test were used to determine statistical difference at *p* < 0.05. Data shown represent the mean ± standard error of the mean (SEM). Data with different letters are considered statistically different. Survival analysis was conducted using Kaplan–Meier estimates (OriginLab Corp., Northampton, MA, USA).

## 3. Results

### 3.1. Piceatannol Blocks Cancer-Associated Lipolysis In Vitro

**Figure 1:**
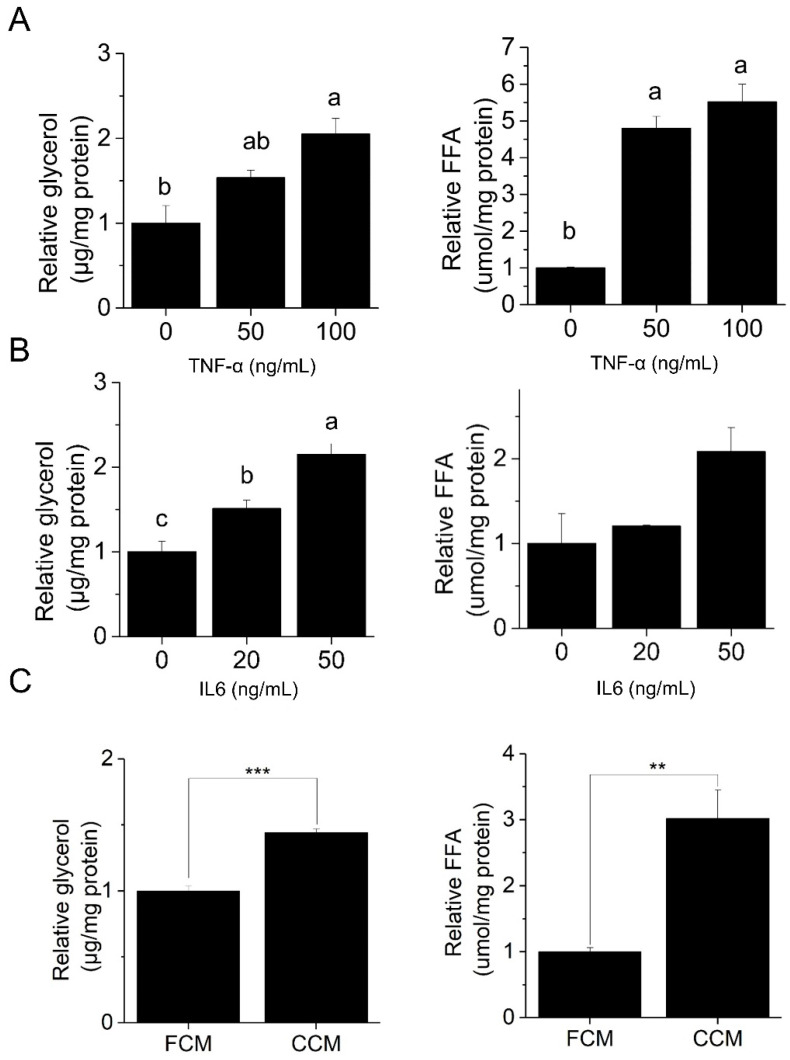
Cancer-associated stimuli increase lipolysis in 3T3-L1 adipocytes, as measured by glycerol and free fatty acid (FFA) release. Mature 3T3-L1 adipocytes were treated with (**A**) TNF-α (16 h), (**B**) IL-6 (16 h), (**C**) NIH-3T3 fibroblast-conditioned media (FCM) or PANC-1 cancer conditioned media (CCM) (8 h), *n* = 3/group. All data presented were mean ± S.E.M. Treatments with different letters are statistically different (*p* < 0.05, Tukey’s post-hoc test following ANOVA). Where only two treatments were analyzed, *p* values were calculated using Student’s *t*-test. ** *p* < 0.01; *** *p* < 0.001.

**Figure 2:**
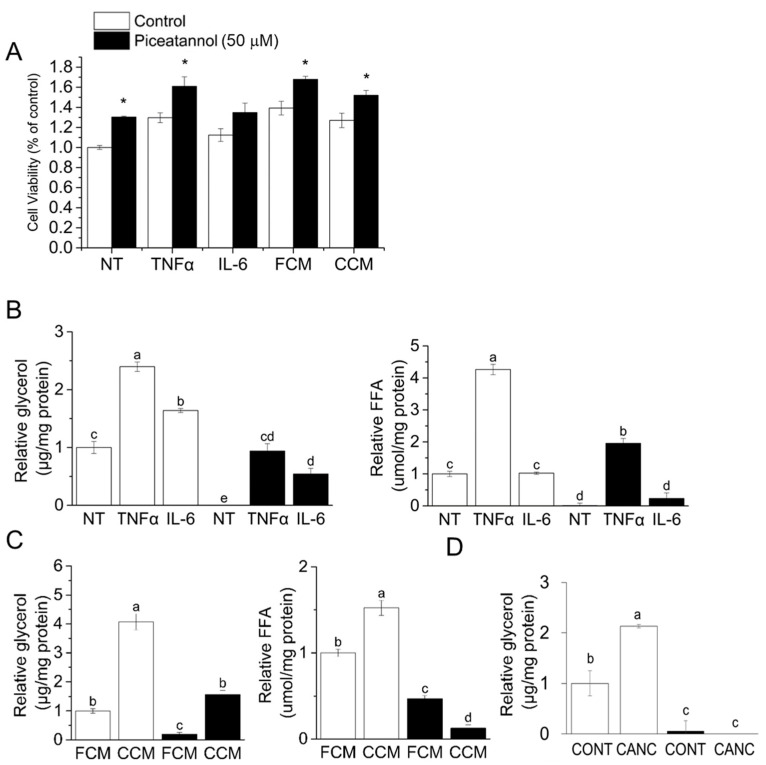
Piceatannol prevents cancer-associated lipolysis. (**A**) Mature 3T3-L1 adipocytes treated with cancer-associated stimuli in the presence or absence of 50 μM piceatannol were subjected to the thiazolyl blue tetrazolium bromide (MTT) assay. Mature 3T3-L1 adipocytes were subjected to (**B**) Tumor necrosis factor alpha (TNF-α) or interleukin -6 (IL-6), (**C**) fibroblast- or cancer-conditioned media, or (**D**) co-culture with either non-transformed human duct epithelial pancreatic cells (HPDE6) (CONT) or AsPC-1 pancreatic cancer cells (CANC) in the absence or presence of piceatannol (50 μM) for 3 h. In A-C, 8-h co-treatment with piceatannol treatment followed 16-h stimuli only treatment. Glycerol and free fatty acids (FFA) in the media were measured as markers of lipolysis, *n* = 3/group. All data presented were mean ± S.E.M. Treatments with different letters are statistically different (*p* < 0.05, Tukey’s post-hoc test following ANOVA). Where only two treatments were analyzed, *p* values were calculated using Student’s *t*-test. * *p* < 0.05.

As lipolysis is the main driver of adipose loss in CAC and we previously revealed an anti-lipolytic effect of piceatannol [30], herein we investigated the role of piceatannol in cancer-associated lipolysis. To examine the cancer-associated lipolysis in vitro, we first confirmed the lipolytic effect of CAC-associated stimuli. The CAC-associated pro-inflammatory cytokines, TNF-α (Figure 1A) and IL-6 (Figure 1B) and CCM from PANC-1 human pancreatic cancer cells (Figure 1C) promoted lipolysis in mature 3T3-L1 adipocytes as judged by an increase in glycerol release and free fatty acids (FFA). Furthermore, we confirmed that the effects of piceatannol and CAC stimulators were not due to decreased cell viability (Figure 2A). In both cytokine-induced and PANC-1-derived CCM-induced lipolysis, an 8-h 50 μM piceatannol treatment reduced glycerol and FFA release by at least 50% (Figure 2B,C). Furthermore, piceatannol also inhibited lipolysis induced by three hours in transwell co-culture of 3T3-L1 adipocytes with AsPC-1 human pancreatic cells (CANC) relative to co-culture with non-transformed HPDE6 (CONT) control cells (Figure 2D). Observations of piceatannol’s inhibitory role in multiple models of cancer-associated lipolysis suggest a role of this phytochemical in CAC lipolysis.

### 3.2. Piceatannol Induces Post-Transcriptional Degradation of ATGL and CGI-58

**Figure 3:**
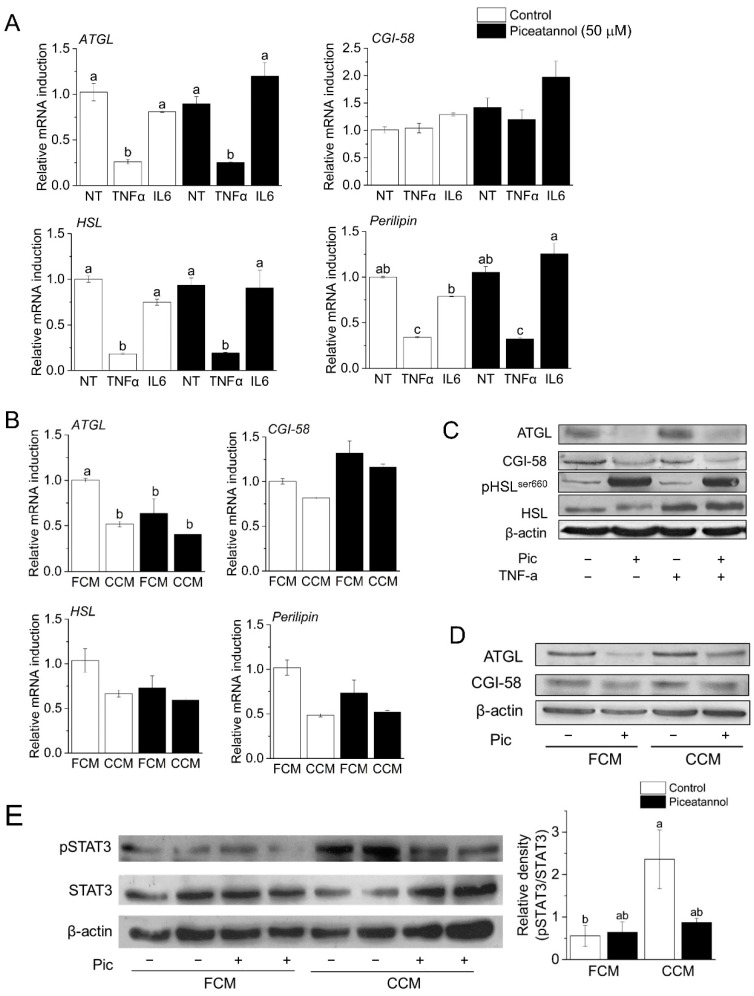
Alterations in expression of lipolytic proteins and genes by piceatannol and cancer-associated stimuli. Mature 3T3-L1 adipocytes were pre-treated with lipolytic stimuli for 16 h followed by 8 h stimuli-piceatannol (50 mM) co-treatment (**A**,**B**,**D**,**E**) or 16 h stimuli-piceatannol co-treatment only (**C**). These cells were subjected to total RNA isolation to determine the mRNA levels of genes involved in lipolysis (adipose triglyceride lipase (*ATGL),* comparative gene identification-58 (*CGI-58)*, hormone-sensitive lipase (*HSL),* and *Perilipin*) (**A**,**B**). Signals were normalized to β-actin (*n* = 3). The expression levels of ATGL, CGI-58, pHSL^ser660^, phosphorylated signal-transducer-and-activator-of-transcription 3 (pSTAT3), and STAT3 in these cells were determined by Western blot analysis using β-actin as a loading control (**C**,**D**,**E**). Values represent relative density of pSTAT3/total STAT3 from three blots. Treatments with different letters are statistically different, as determined by ANOVA followed by Tukey’s post-hoc analysis (*p* < 0.05). All data presented were mean ± S.E.M.

Our recent study demonstrated that piceatannol induced degradation of ATGL and its co-activator CGI-58 in an autophagy-dependent manner [30]. Therefore, our next objective was to verify whether a similar mechanism mediated piceatannol action in models of CAC. In support of piceatannol’s post-transcriptional regulation of lipolytic machinery, we found no difference in mRNA expression of *ATGL, CGI-58, HSL*, and *perilipin* in TNF-α, IL-6, and CCM-treated adipocytes (Figure 3A,B). Consistent with our previous findings, piceatannol induced ATGL and CGI-58 degradation in both TNF-α and CCM stimulated conditions (Figure 3C,D). On the other hand, we found that piceatannol increased the pro-lipolytic serine 660 phosphorylation of HSL, a key event in HSL activation [34] (Figure 3C). As the inflammatory cytokine IL-6 mediates adipose loss in various *in vivo* models of CAC [35], and piceatannol is suggested to inhibit IL-6 signaling and secretion and its associated signal-transducer-and-activator-of-transcription 3 (STAT3) signaling pathway [36,37], we determined the effect of piceatannol on CAC-induced STAT3 activity in adipocytes. We observed that piceatannol suppressed STAT3 phosphorylation in CCM-treated adipocytes, although the difference was not statistically significant (p = 0.100) (Figure 3E). Taken together, our results support the hypothesis that piceatannol inhibits lipolysis via post-transcriptional modification of ATGL and CGI-58.

### 3.3. Piceatannol Slows Weight Loss and Wasting in Tumor-Bearing Mice by Preserving Adipose Tissue Mass

**Figure 4:**
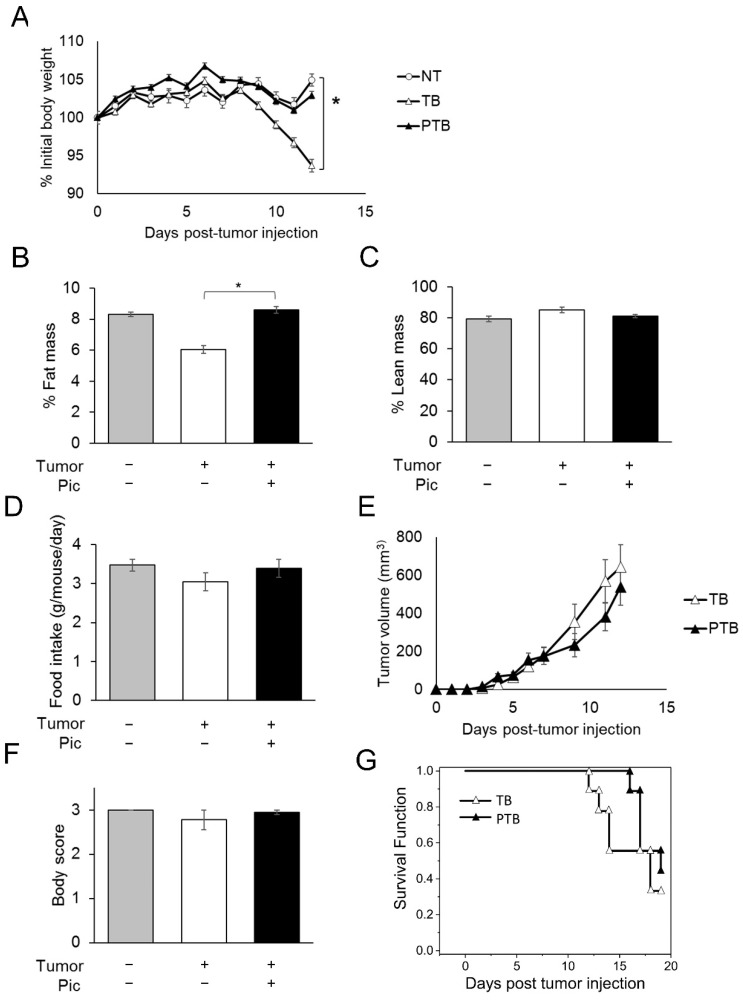
Piceatannol affects body weight independent of tumor growth and food intake. Non-tumor bearing (NT, *n* = 4) or C26 colorectal carcinoma tumor-bearing (TB, *n* = 9) male BALBc received a vehicle or piceatannol (PTB, *n* = 9) beginning on day 5. (**A**) Body-weight, (**B**) fat mass, (**C**) lean mass, (**D**) average daily food intake, (**E**) tumor volume (**F**), and body score (**G**) are shown. (**G**) The Kaplan–Meier estimator of survival of the mice in TB and PTB. All data presented were mean ± S.E.M. *p* values were calculated using Student’s *t*-test. *, *p* < 0.05.

As lipolysis is known to contribute to rapid fat loss and overall body weight loss in CAC, we next investigated whether piceatannol’s anti-lipolytic function could protect C26 carcinoma-bearing mice from cancer-induced body weight and adipose loss. Consistent with previous reports [32,38], tumor-bearing (TB) mice started to lose body weight 8 days post-tumor injection (Figure 4A). By Day 12, TB mice experienced approximately 10% decrease in the percentage of initial body weight and weighed significantly less compared with non-treated control (NT) mice. However, piceatannol-treated tumor-bearing (PTB) mice receiving 20 mg/kg BW/day (*i.p.*) displayed no difference in body weight from NT mice (Figure 4A). At the end of the study (Day 12), the average body weights of mice in NT, TB, and PTB were 25.3 g, 22.8 g, and 24.4 g, respectively. Furthermore, body composition analysis by Echo magnetic resonance imaging (MRI) [39] revealed that TB mice had 31.6% less fat mass than NT mice, while piceatannol treatment preserved body fat mass in PTB mice (Figure 4B). A significant difference in percent fat mass, but not percent lean mass in TB mice (Figure 4C) suggests that piceatannol protected body weight primarily through preservation of adipose tissue. No difference was detected in food intake (Figure 4D) and tumor growth (Figure 4E) across all groups, suggesting that the observed effect was not due to an indirect effect of piceatannol on energy intake or tumor mass. Interestingly, changes in body weight and adipose mass were observed prior to significant changes in the appearance of wasting, as indicated by an average body score near the optimal condition score of 3 (Figure 4F). We next tested the effect of piceatannol on survival of tumor-bearing mice. In line with the tumor growth result (Figure 4E), survival analysis revealed that piceatannol-treatment did not significantly alter the overall survival rate of mice in TB and PTB groups, despite a suggestive trend (Figure 4G).

## 4. Discussion

In this study, we revealed a novel role of a dietary compound, piceatannol, in blunting cancer-associated lipolysis in vitro, and adipose and body-weight loss in a mouse model of cachexia. Despite increasing interest in natural anti-cancer compounds, little is known about dietary modulation of cancer-associated adipose wasting. Although others have demonstrated a role of naturally occurring bioactive compounds, such as alantolactone [40], nicotinamide [41], coix seed oil [42], and carnosol [43], in slightly alleviating fat loss, our study is the first report, to our knowledge, of a dietary compound exhibiting protection of adipose tissue in early stages of CAC. These results add needed evidence to support the protective role of dietary compounds generally and piceatannol specifically in preserving adipose tissue and lowering lipolysis in models of CAC. 

Adipose lipolysis in general, and ATGL specifically, are considered key targets to preserve body weight in CAC [14,17,44]. For example, tumor-bearing ATGL knockout mice are protected from both adipose and muscle loss, ATGL has increased expression in mouse models of CAC, and ATGL activity negatively correlates with body mass index in CAC patients [7,17,35]. Although ATGL inhibitors have been developed for the treatment of CAC [45,46], they have not yet been implemented in clinical trials [44]. Here, we demonstrate that piceatannol acutely blunted lipolysis and lowered ATGL and CGI-58 protein levels with little effect on their mRNA levels in various cancer-associated conditions. Importantly, piceatannol reduced glycerol release in TNF-α-stimulated lipolysis, despite activation of HSL seen by Ser660 phosphorylation, suggesting the inability of other lipases to compensate for ATGL loss. Together with others who have demonstrated an inability of HSL to fully compensate for ATGL inhibition, our study adds further evidence of the central role of ATGL in CAC lipolysis [47]. Taken together, these findings reveal a novel function of piceatannol in regulating a central lipolytic pathway in models of CAC, in that piceatannol targets lowering protein levels of the key lipolytic enzymes ATGL and CGI-58.

Increasing evidence demonstrates that adipose wasting occurs before muscle loss in the early stage of CAC and preserving adipose mass early in CAC represents a promising therapeutic strategy [7,13]. Slowing weight loss in CAC can improve quality of life and increase survival outcomes in cancer patients, further emphasizing the need for weight-stabilization strategies [4,10]. Consistent with other studies, we found that body weight loss was driven primarily by adipose depletion in earlier stages of CAC [48]. Piceatannol-treated mice, however, were completely protected from adipose loss in early CAC as judged by an accurate analysis of body composition by EchoMRI. Despite preserving adipose in the initial stages of CAC, piceatannol treatment did not significantly prolong survival; this finding is consistent with others’ observation of lipolysis inhibition and adipose preservation without improved survival in similar models of CAC [25]. While adipose loss may be a key feature of early cachexia, our results indicate that preventing WAT loss alone may be insufficient to prolong survival. Although piceatannol has been previously reported to suppress tumor progression in various cancer types such as mammary cancer [49], ovarian cancer [50], and prostate cancer [51], we observed no effect of piceatannol on tumor size. This discrepancy could be explained by a cancer-specific efficacy of piceatannol and/or variation in piceatannol treatment regimens. Despite limited evidence from the current study concerning a beneficial effect on survival or tumor size, piceatannol, due to its adipose-preserving properties, could be used in combination with therapies that reduce tumor burden or preserve lean muscle mass. Indeed, strategies to preserve body weight early in CAC are needed to complement other anti-cancer therapies [52,53]. Thus, investigations of piceatannol’s complementary effect on anti-cancer therapies merit further research.

Further investigation of the mechanism by which piceatannol preserves adipose tissue is needed to better understand its therapeutic potential, as this study focused on “proof of concept” of piceatannol’s anti-lipolytic role in in vitro models of CAC and adipose preservation *in vivo*. Notably, we recently demonstrated that piceatannol, unlike resveratrol, plays an inhibitory role in both basal and stimulated lipolysis in cultured adipocytes, and diet-induced obese mice [30]. We further found that piceatannol-inhibited lipolysis was mediated by degradation of ATGL, CGI-58, and perilipin1 proteins through activation of autophagy-lysosome pathway in adipocytes. Thus, future study should focus on investigating the molecular basis underlying piceatannol-regulated lipolytic protein clusters in adipocytes under CAC conditions. Moreover, future studies could investigate the role of piceatannol’s anti-inflammatory properties in addressing CAC, as inflammation plays a significant role in the progression of CAC [54]. Studies on piceatannol’s effect in other models of CAC are also needed to more fully establish its protective properties. As causes of CAC are complex and not completely understood, multiple approaches are needed to establish causal relationships of therapeutic interventions. Furthermore, because piceatannol was administered interperitoneally in our study, studies using oral administration of piceatannol would further inform optimal therapeutic strategies. Such studies would lay the groundwork to determine doses of piceatannol needed to achieve clinical significance, and whether meaningful doses could be obtained with dietary or supplemental interventions.

## 5. Conclusions

In summary, we provide evidence of a protective role of piceatannol, a natural compound, in attenuating cancer-associated wasting. Using various in vitro models, we showed that piceatannol treatment is sufficient to block pro-lipolytic CAC-associated stimuli. Furthermore, we confirmed that piceatannol induces ATGL degradation, an important target to preserve body weight in CAC [17]. Although the causes of adipose loss in CAC are complex, it is becoming increasingly apparent that downstream signaling effectors of lipolysis, i.e., lipases, are of key importance [55]. Piceatannol’s anti-lipolytic properties may translate to *in vivo* models, as piceatannol preserved adipose tissue in early stages of cachexia. Synergistic effects of piceatannol with other anti-cancer therapies in CAC paradigms merit further investigation.

## Data Availability

Not applicable.
